# Assisting informed decision making for labour analgesia: a randomised controlled trial of a decision aid for labour analgesia versus a pamphlet

**DOI:** 10.1186/1471-2393-10-15

**Published:** 2010-04-08

**Authors:** Camille H Raynes-Greenow, Natasha Nassar, Siranda Torvaldsen, Lyndal Trevena, Christine L Roberts

**Affiliations:** 1 Kolling Institute of Medical Research, University of Sydney, Sydney, NSW, Australia; 2 Sydney School of Public health, University of Sydney, Sydney, NSW, Australia

## Abstract

**Background:**

Most women use some method of pain relief during labour. There is extensive research evidence available of pharmacological pain relief during labour; however this evidence is not readily available to pregnant women. Decision aids are tools that present evidence based information and allow preference elicitation.

**Methods:**

We developed a labour analgesia decision aid. Using a RCT design women either received a decision aid or a pamphlet. Eligible women were primiparous, ≥ 37 weeks, planning a vaginal birth of a single infant and had sufficient English to complete the trial materials. We used a combination of affective (anxiety, satisfaction and participation in decision-making) and behavioural outcomes (intention and analgesia use) to assess the impact of the decision aid, which were assessed before labour.

**Results:**

596 women were randomised (395 decision aid group, 201 pamphlet group). There were significant differences in knowledge scores between the decision aid group and the pamphlet group (mean difference 8.6, 95% CI 3.70, 13.40). There were no differences between decisional conflict scores (mean difference -0.99 (95% CI -3.07, 1.07), or anxiety (mean difference 0.3, 95% CI -2.15, 1.50). The decision aid group were significantly more likely to consider their care providers opinion (RR 1.28 95%CI 0.64, 0.95). There were no differences in analgesia use and poor follow through between antenatal analgesia intentions and use.

**Conclusions:**

This decision aid improves women's labour analgesia knowledge without increasing anxiety. Significantly, the decision aid group were more informed of labour analgesia options, and considered the opinion of their care providers more often when making their analgesia decisions, thus improving informed decision making.

**Trial Registration:**

Trial registration no: ISRCTN52287533

## Background

Many factors are considered influential in determining women's experience and satisfaction with childbirth. Women's expectations of the duration and level of pain suffered, quality of her care-giver support, and involvement in labour decision making are the most commonly reported factors[1].

In developed countries most women use some method of pain relief during labour. Significantly, there have been more clinical trials of pharmacological pain relief during labour and childbirth than of any other intervention in the perinatal field[2]. However to what degree this evidence is available to pregnant women is unclear.

The importance of discussing women's preferences for labour pain relief before labour begins is well established although it may not be well practiced[2]. A survey of Australian women found that antepartum information about analgesia was most commonly derived from hearsay and least commonly from health professionals[3]. A study conducted in the UK found that most women want more detailed and specific information about pain relief in labour[4], and a survey of 790 Australian women reported a tenfold increase in dissatisfaction among women who did not have an active say in decisions about their pregnancy care[5]. Similarly women in another study rated the explanation of procedures, including the risks, before they are carried out and involvement in decision making as most important in their satisfaction with care[6]. A recommendation from a US survey suggested that women need access to results of the best available research about the effectiveness and possible side-effects of both pain medications and drug-free measures [7], and a recent systematic review suggested that there is a gap between women's expectations and their actual experiences[8].

Decision aids are interventions designed to help people make specific and deliberative choices among options by providing (at minimum) information on the options and outcomes relevant to the person's health status[9]. Decision aids differ from usual health education materials because of their detailed, specific and personalised focus on options and outcomes for the purpose of preparing people for decision making. They have been widely used in a variety of health settings and a Cochrane systematic review of decision aids identified over 200 aids. Only a few decision aids and a series of leaflets have been developed and tested specifically for pregnancy and birth issues [10-14], despite this being an area in which consumers are known to want to actively participate in decision making[5].

We developed a decision aid for labour analgesia for primiparous women planning a vaginal delivery. The aim of the decision aid was to reduce decisional conflict (uncertainty regarding which option to use)[15], increase labour analgesia knowledge, without increasing anxiety and increase satisfaction with decision making in regards to labour analgesia. We tested the effectiveness of the decision aid in a randomised controlled trial.

## Methods

### Study Setting

The trial was conducted in Sydney, Australia in two obstetric hospitals between September 2004 and April 2006, in accordance with the published protocol [16]. One hospital was a tertiary public hospital the other a private hospital. In Australia, women can choose to have pregnancy care in a public hospital which is covered by the national health insurance, or choose private care by an obstetrician in a public or private hospital. Public care is usually provided by hospital midwives overseen by salaried doctors. Public patients choose between the midwife antenatal clinic, the midwife run birth centre or use 'shared care' (joint antenatal care between the hospital antenatal clinics and their own general practitioner). In the private hospital all women receive private antenatal and perinatal care from their chosen obstetrician. Both trial hospitals provided a range of non-drug and anesthesia options for pain relief in labour. Epidurals were available 24 hours a day from anesthesia staff designated to labour ward in both hospitals. Epidural rates in the trial hospitals for women with a singleton term infant, with labour and a vaginal birth were approximately 26%. Rates in the private hospital for the same group of women were higher, closer to 50%.

### Participants and eligibility criteria

Primiparous women, in their final trimester, who were planning a vaginal birth of a single infant, were eligible for the study. Primiparous women were selected because previous pregnancy has a strong impact on decision making and analgesia use in labour[17]. Excluded women were those who did not have a choice regarding their analgesic options (for known preexisting conditions), had contraindications to analgesia, or had self-assessed English insufficiency (insufficient to complete the questionnaires, or use the intervention material which were written in English).

### Intervention

We developed a labour analgesia decision aid that presented information in two formats: booklet only, and booklet plus audio guide.

The decision aid material was developed and pilot tested using an iterative process of review and revision with a multidisciplinary group, including consumers and was based on the Ottawa Health Decision Group method[18]. The decision aid content (the booklet and the audio text) was assessed using the Flesch-Kincaid Grade and graded 9.9 demonstrating that is was accessible to the average ninth grade student. The final booklet was approximately fifty-five, A5 pages and was accompanied by a 4-page A3 worksheet. The audioguide was a ~40-minute audio compact disc (CD). Information was presented in a style that was very sparse so that it was user friendly and only one analgesic option was presented per two page spread. The decision aid and worksheet can be found at http://www.psych.usyd.edu.au/cemped/com_decision_aids.shtml.

The content of the decision aid included a wide range of both pharmacological and non-pharmacological analgesics that were used and accepted in the trial hospitals. Evidence for each option was based on the highest quality of research evidence available, in most cases this was based on systematic reviews with meta analyses (Table 1). The two main outcomes presented for each analgesic option were women's satisfaction with the pain relief and need for further pain relief after using the analgesic method. Probabilities of other outcomes for the mother and the baby were synthesised from the evidence and were presented as a list of pros and cons for each analgesic option.

**Table 1:** Labour and childbirth analgesic options included in the decision aid and the evidence base

Analgesic option	Evidence base
Support person	Systematic review[32]
Being upright during labour	Three systematic reviews [33-35]
Touch and massage	Two systematic reviews, one based on observational data[36]
Bath	Systematic review[37]
Aromatherapy	Systematic review[37]
Acupuncture	Systematic review[37]
Hypnosis	Systematic review[37]
Transcutaneous Electrical Nerve Stimulation (TENS)	Systematic review[38]
Nitrous oxide	Systematic review[39] and population based data[40]
Opioid analgesia	Two systematic reviews[41,42] and population based data[40]
Epidural analgesia	Two systematic review[43,44] and observational research [45].

### Standard Care

Information about analgesia is widely available to women from many sources including their antenatal care providers, antenatal classes, books, friends, family and the internet, but there is no standard care per se[19]. As it was difficult to define 'standard care' in regards to analgesia information, women randomised to standard care in this trial received a pamphlet called "Pain relief during childbirth - A guide for women". It was a 4 page A3 pamphlet presenting information and risk data for both pharmacological and non-pharmacological labour analgesia, and was developed and endorsed by The Royal Australian and New Zealand College of Obstetricians and Gynaecologists, and the Australian Society of Anaesthetists.

### Procedures

The trial was conducted as a three arm trial with the two decision aid arms (an audio-guided decision aid versus a non-audio decision aid) and a third arm who received the pamphlet. As stated in the protocol, originally we designed a two arm trial. We added a third arm to test the effectiveness of an audio component in a decision aid as this had not been previously done. Our analysis plan was to test any differences in decisional conflict between the two decision aid groups and if there were no differences combine the two decision aid groups for the analyses for this manuscript. The results of the prespecified comparison of these two decision aids found that there were no differences in outcomes between the two presentation formats (audio-guide decision aid mean decisional conflict score 24.32, compared to booklet decision aid 23.56, mean difference 0.7, (95%CI (-1.4, 2.9))[20] and as planned the two decision aid groups were combined for these analyses. This resulted in differences in the size of the two arms.

**Figure 1:**
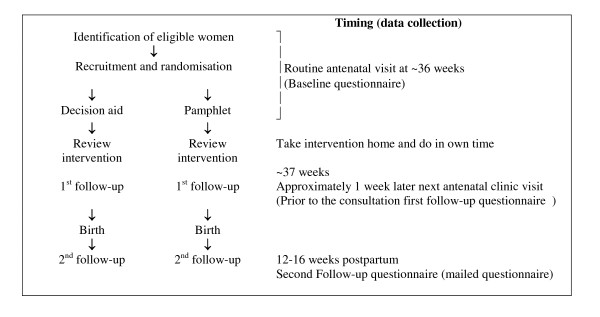
**Trial Schema**.

Using a pragmatic approach the trial protocol utilised the usual schedule of weekly antenatal visits in late pregnancy, this was to assess the decision aid under the conditions most likely to be applied in practice (Figure 1).

Women were randomly allocated via a remote location, to one of the study groups and baseline data were collected. They then received the trial materials and worked through this on their own. At their next appointment (the following week) any questions were discussed with the research assistant and the first follow-up questionnaire was completed. At three months postpartum, a second follow-up questionnaire was mailed with reply paid envelopes to all participants. Information on pregnancy and birth outcomes was obtained from the hospital obstetric records.

Treatment allocation was randomly generated by computer using random variable block sizes http://www.randomization.com. It was not possible to conceal allocation once randomised; however to minimise contamination a number of methods were utilised [15] (also see Bias section below).

### Outcome Measures

The effectiveness of the decision aid to improve women's decision-making was determined by assessing primary outcomes of knowledge of labour analgesia, decisional conflict (uncertainty regarding analgesia decision) and anxiety. These were measured using self-administered questionnaires that have been extensively used and validated in decision aid analysis and were adapted for this content where necessary[15,21,22]. The question format was based on the style of the Ottawa Health decision group, and on our own previous work [15] adapted for this context and are described in more detail in the following section.

### Knowledge

Knowledge of labour analgesia was assessed by asking women true/false questions at baseline and at first follow-up. The measure included 16-questions related to general knowledge about labour analgesia risks and benefits. The content was based on general knowledge about labour analgesia, which is typically available and designed for the general public. It did not require any specialist knowledge and would be an appropriate level for gaining informed consent.

### Decisional conflict scale

Decisional conflict refers to uncertainty about a course of action and in this case was related to the decision of what analgesia to use in labour [15]. The scale measures the constructs of uncertainty and factors contributing to uncertainty (such as feeling uninformed, unclear about values, and unsupported in decision making). The scale is reliable, discriminates between those who make or delay decisions, and is sensitive to change[9,20,21]. Each item was scored according to the instructions and standardised to a score between 0 representing low decisional conflict, and 100 extreme decisional conflict. A low score (<25) indicates a low decisional conflict.

### Anxiety

Anxiety was measured using the state component of the short Spielberger anxiety scale[23]. It is reliable and has been validated for use with decision aids. The short form is a 6-item scale, and scores are range from 20 to 80, the higher the score the greater the level of anxiety.

### Stages of decision making

Measuring how receptive people are to changing their mind regarding their labour analgesia choices was measured using the 'stages of decision making scale'. It has been widely used in decision aid trials[9]. It is a nominal ordinal scale from 1 to 6, where 1 is "Haven't begun to consider choices" and 6 is "Unlikely to change my mind". It gives an indication as to what stage of the decision making women are in, and also indicates how receptive women are to changing their choices in regards to their decisions. Women using decision aids should be between stage 2 and 5, and ideally between stages 2 and 3 which are considered active deliberation stages of decision making.

### Satisfaction with decision making

Satisfaction with the analgesia decisions was measured using a validated six-item scale, satisfaction with the decision (SWD). The SWD scale predicts decision certainty. It correlates with the likelihood of the patients intentions to use a health intervention[9,22].

### Choice predisposition: analgesia preferences and use

In trials of decision aids preferences or uptake of options are measured as the percentage of individuals stating a preference for or actually implementing the most intensive or most invasive option[9]. For this trial choice predisposition was measured at baseline and at follow-up 1 after the intervention. Intentions or plans to use analgesia were measured using a five point nominal scale from 'no plans to use' to 'very definite plans to use'. Actual use of analgesia was measured using self-report (which was validated by hospital records). The proportion of women who reported 'no plans to use', or 'definitely not going to use' an analgesic option were compared to their actual use. This was used as a measure of decision follow-through and uptake of options.

### Participation in decision making

Participation in decision making was measured by the Control Preferences Scale[21]. The scale measures the preferred or actual role in decision making using five response statements - two responses represent an active or self controlled role, one response equals a shared or collaborative role, and the final response statements represents a passive or practitioner controlled role.

### Adherence and acceptability

Data were collected on monitoring adherence with the intervention, i.e. had the women actually used the decision aid (read all, some or none or it), listened to the CD and completed the worksheet. General comments regarding the intervention, acceptability, if women felt they had enough information to make labour analgesia decisions and whether women would recommend the materials was also ascertained.

### Assessing the impact of the decision aid on services/outcomes

To assess the impact of service utilisation data were extracted from the routinely collected hospital database. Data on analgesia use and maternal and perinatal outcomes were collected. Data included marital status, smoking, maternal age, parity, onset of labour, mode of birth (vaginal, planned caesarean section or caesarean section after the onset of labour.

### Statistical issues

#### Sample size

Based on data from one of the trial hospitals, about 2300 primiparous women gave birth to singleton infants after 36 weeks gestation of which approximately 94% used some form of analgesia. It was anticipated that at least 50% of women would be both eligible and willing to participate. Based on a meta-analysis[9](data from the 2002 version) comparing decision aids to a pamphlet reporting a pooled mean difference for decisional conflict of -4.35 (95%CI - 6.8, -1.9) we calculated a sample size of 141 women in each arm. To change knowledge scores only 25 women in each arm were required however we planned to change decisional conflict if possible and hence chose the larger sample size calculation. The sample size was further inflated to allow for loss to follow-up and we determined that we required 195 women in each arm (significance 0.05, power 0.8).

#### Data analysis

Analyses were by intention to treat. At baseline, univariate analyses of demographic variables, analgesia knowledge, decisional conflict and anxiety were carried out between study groups. Univariate analyses were further performed for primary and secondary outcomes. Results for knowledge outcomes were analysed by summing and calculating the percentage of correct responses for each individual. Scoring for affective outcomes measures were calculated according to recommended algorithms[9,21,22]. Group differences in categorical outcome variables were assessed using Chi-squared or Fisher exact tests. Yates' correction was applied to tables with one or more cells with frequencies less than five[24]. Continuous variables were examined using the 2-sample t-tests using Satterwaite correction in cases with unequal variances[24]. For repeated measures, analysis of variance was conducted to assess group differences in outcomes over time. Two-sided p-values less than 0.05 were statistically significant and all data were analysed using SAS version 9.1 (SAS Institute, Cary, NC). Missing data was not included.

An a priori sub-group analysis was also conducted to exclude women who lost their options for labour analgesia and which may have impacted on their satisfaction, anxiety and decisional conflict outcomes. Thus women who had a planned caesarean section after randomisation, or women with caesarean section without labour, an emergency caesarean section or an epidural for therapeutic reasons were excluded from these subgroup analyses.

Our results are presented temporally. Initially we present the baseline comparisons between the groups. These are followed by the first follow-up results of the primary and secondary outcome measures and then these same measures but at the second-follow-up.

#### Ethical considerations

The study was approved by the Central Sydney Area Health Service Ethics Review Committee (Protocol no.# X02-0247) and the University of Sydney Human Ethics Committee (Ref No. 3419).

#### Bias

As this was an unblinded trial we used various methods to reduce bias. For selection bias: eligible women were identified from the patient appointment book each morning, and to the best of our ability most of these were approached, although this is not usually an issue for RCT's. We also used remote telephone randomisation. For observation/information bias we used: data collection forms based on standardised instruments that used highly objective closed ended questions, researchers were kept blinded to women's intervention allocation as much as possible, the research assistant followed an interview protocol at each follow-up, and had been trained in the implementation of keeping the follow-up standardised regardless of intervention allocation. Usual antenatal care providers were blinded to the content and format of the decision aid, regular in-service (educational training) was conducted for the antenatal care providers to explain the trial protocol and to make clear the potential effect of unmasking or contamination, and most women who received the pamphlet were unaware that it was not the intervention. So although it was not possible to truly blind women in the trial we made every effort to blind.

## Results

### Participant follow-up

**Figure 2:**
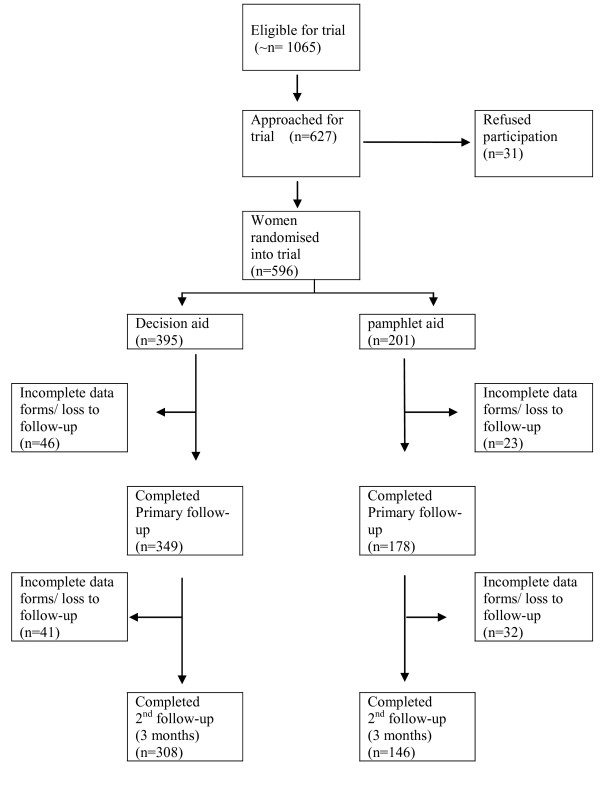
**Flow of participants through trial**.

Between September 2004 and February 2006, 596 women were randomised into the trial, of these 395 received the decision aid and 201 received the pamphlet (Figure 2). At first follow-up (primary outcomes) the overall response rate was 88%, and there were no significant difference between groups (χ^2 ^= 0.12 df = 1, p = 0.827). The overall response at second follow-up was 78% and was not significantly different between groups (χ^2 ^= 1.41 df = 1, p = 0.234).

### Baseline measurement

#### Demographic measures

As seen in Table 2, maternal demographic characteristics and baseline measures of cognitive and affective outcomes were comparable between these two groups.

**Table 2:** Baseline maternal characteristic of the decision aid group and the pamphlet group

Maternal Characteristics	Decision Aid (n = 395) n (%)	Pamphlet (n = 201) n (%)
Maternal Age [mean years] (range)	30.9 (21 - 44)	30.7 (19 - 44)
Gestational age at recruitment [mean weeks] (range)	35.8 (28 - 40)	36.0 (30 - 38)
Education		
Primary school	18 (4.6)	12 (6.0)
High School	45 (11.4)	24 (11.9)
Technical/other	100 (25.3)	59 (29.4)
University	232 (58.7)	106 (52.7)
Marital status		
Living with partner	377 (95.2)	185 (92.1)
Not living with partner	18 (4.8)	16 (7.9)
Type of obstetric care		
Public	347 (87.9)	176 (87.7)
Private	44 (11.2)	23 (11.5)
*n missing (%)*	4 (1.1)	2 (1.0)
Smoking status at first antenatal appointment [self-report]		
Current smoker	21 (7.4)	12 (9.3)
Ex-smoker	38 (13.4)	16 (12.4)
Never smoked	336 (79.0)	173 (78.2)
Proportion who had 'definite' plans to use		
Support person	300 (87.0)	152 (81.7)
Bath	147 (38.7)	76 (39.9)
Nitrous Oxide	72 (18.7)	36 (18.5)
Opioid analgesia Epidural analgesia	10 (2.6) 28 (7.1)	6 (3.2) 18 (9.0)
		
Knowledge mean score (SD)	53.4 (21)	54.3 (20)
[% correct responses]		
*n missing (%)*	*14 (3.5)*	*4 (2)*
		
Decisional Conflict mean score (SD)	31.4 (12.8)	31.2 (13.4)
[0-100, 0 = very low decisional conflict]		
*n missing (%)*	*9 (2.8)*	*2 (1)*
		
Anxiety mean score (SD)	34 (10)	34 (11)
[20-80, 20 = low anxiety]		
*n missing (%)*	*5 (1.2)*	*2 (1)*

Per cent may not add up to 100 due to rounding.

Most women were in the last weeks of pregnancy as per the study protocol. A large proportion of both groups were university educated (decision aid group 59% and pamphlet group 53%) and almost all lived with their partner (either married or co-habiting, decision aid group 95% or pamphlet group 92%).

#### Knowledge, decisional conflict and anxiety measures

Baseline measures of the primary outcomes were identical between the two groups. Most women had only a moderate knowledge level of labour analgesia. Overall the median decisional conflict scores suggested mild decisional conflict, and were not different between groups. Similarly the anxiety scores were not different between groups and were suggestive of slightly elevated anxiety.

#### Analgesia intentions

Baseline intentions for analgesia use were comparable between the two groups. Overall higher proportions of women in both groups intended to use non-pharmacological methods of pain relief for labour than pharmacological methods. Approximately 5-8% of women were definitely intending to have an epidural for labour. The majority of participants had definite intentions to have a support person with them during labour, and approximately 40% of women planned to use a bath during labour.

#### Stages of decision making

At baseline we assessed what stage in labour analgesia decision making women were in. There was an even distribution of stages of decision making between the two groups. A very small proportion of women in both groups were not considering their choices (decision aid 3.3% versus pamphlet 2.5%), or had made up their mind and were 'unlikely to change mind' 17% in both groups. However a large proportion of women in both groups were in stages that were amenable to change or were in active deliberation stages (decision aid 25.2% versus pamphlet 21.6%). The largest proportion in both groups were women who 'had made some choices but were willing to reconsider' (decision aid 54.3%, pamphlet 58.3%).

## Post intervention - First follow-up, ~38 weeks gestation

### Knowledge, decisional conflict and anxiety measures

At first follow-up when the primary outcomes were measured, there was a significant difference in knowledge scores between the decision aid group (mean score 65%) compared to the pamphlet group (mean score 56%) (mean difference 8.6, 95% CI 3.7, 13.4). This was consistent for all subscales within the knowledge questionnaire (not reported).

After using the interventions the level of decisional conflict for both groups decreased, however there was no difference between groups (decision aid 23.94 compared to pamphlet group 24.93) (mean difference -0.99 (95% CI -3.07, 1.07). On this scale, scores in this magnitude are usually associated with decision follow through, indicating low decisional conflict.

There were no significant differences between anxiety scores of the two groups at primary follow-up (mean difference 0.3, 95% CI -2.15, 1.5). The mild anxiety scores of both groups measured at primary follow-up were unchanged from the baseline measurement.

### Satisfaction with analgesia decision making

At first follow-up both groups were equally and highly satisfied with the labour analgesia plans that they had made (Table 3).

**Table 3:** Cognitive, affective and behavioural outcomes

Cognitive, affective and behavioural outcomes	Decision aid (n = 395)*	Pamphlet (n = 201)*	Mean difference (95%CI)	P-value
Decisional conflict (1-100, 1 = low decisional conflict)				
Baseline	31.4 (12.8)	31.2 (13.4)	0.22 (-2.0, 2.7)	0.84
Primary follow-up	23.9 (10.6)	24.9 (12.9)	-0.99 (-3.1, 1.1)	0.37
Second follow-up	19.9 (12.3)	20.2 (14.1)	-0.31 (-2.9, 2.3)	0.82
				
Knowledge (% correct responses)				
Baseline	53.4 (21.9)	54.4 (20.9)	-0.94 (-4.6, 2.7)	0.61
First follow-up	65.1 (29.5)	56.5 (27.4)	8.58 (3.7, 13.5)	<0.01
				
Anxiety (20-80, 20 = low anxiety)				
Baseline	33.9 (10.1)	34.3 (11.8)	-0.32 (-2.2, 1.5)	0.74
First follow-up	33.3 (9.3)	34.3 (11.0)	-0.96 (-2.8, 0.8)	0.32
Second follow-up	29.4 (8.5)	29.0 (9.5)	0.55 (-2.3, 1.2)	0.54
				
Satisfaction with decision making [% satisfied]				
First follow-up	81.5 (10.3)	80.7 (11.7)	0.86 (-1.1, 2.9)	0.40
Second follow-up	84.4 (12.9)	82.8 (16.1)	1.53 (-1.9, 4.4)	0.32
				
Enough information to make decision [%]				
1st follow-up	89.2	79.8	RR 1.34 (1.1, 1.7)	<0.01
				
Participation in decision making [%]1st follow-up				
I will choose by myself	76.7	80.6		
Shared decision with care-providers	22.1	17.7	RR 0.93 (0.8, 1.0)	
Care-providers decides	1.2	1.7		
				
Participation in decision making [%]2nd follow-up				
I chose by myself	34.5	49.6		
I chose after seriously considering my care- providers opinion	37.8	30.7	= 9.4	0.09
Shared decision with care-providers	19.3	13.8		
Care providers made decisions	6.0	4.9		
Other	2.7	1.5		

*Numbers may not add up to totals due to missing data

There was a significant difference between the groups (p = 0.009) and whether they considered they had enough information to make decisions regarding labour analgesia (Table 3). This was significantly higher amongst women in the decision aid group (89.2%) compared to the pamphlet group (79.8%) (RR 1.34, 95% (CI 1.06, 1.69)).

### Control preference scale

There were no differences between groups of who should participate in their labour analgesia decision making (RR 0.93 95%CI 0.81, 1.07). Overall 98% of women regardless of the trial group wanted to be actively involved in their labour analgesia decision making. The majority of women in both groups were planning to make their decisions by themselves, without necessarily consulting their care providers (Table 3).

### Source of labour analgesic information, adherence and acceptability

Women were asked from where they had received their labour analgesia information. There were no significant differences between groups and their information sources. Both groups equally relied on family and friends, books and antenatal classes.

Women in both groups were asked about their use, adherence and acceptability of the intervention they received and most women responded they had read all of the intervention (decision aid 98% compared pamphlet group 95%, χ^2^= 2.782, df = 1, p = 0.061), and equally both groups would recommend the intervention they received to a pregnant friend (decision aid group 94% compared pamphlet group 93%, χ^2 ^= 0.33, df = 1, p = 0.57).

## Second follow-up, 12-16 weeks post-partum

### Pregnancy, labour and birth outcomes

There were no differences between labour and birth outcomes between the groups (Table 4). Approximately half of the women experienced a spontaneous labour, and almost one third had labour induced. The majority of women had a vaginal delivery (~75%), of those twenty per cent had an instrumental vaginal delivery.

**Table 4:** Pregnancy, labour and birth outcomes by group.

Pregnancy and birth outcomes	Decision aid group (n = 395) n (%)	Pamphlet group (n = 201) n (%)	p-value
Labour			
Spontaneous	225 (63)	119 (68)	
Induced	115 (32)	42 (24)	
No Labour	15 (4)	11 (6)	0.97
			
Mode of delivery			
Vaginal (non-instrumental)	201 (57)	97 (56)	
Instrumental delivery	72 (20)	34 (19)	
Caesarean section	82 (23)	41 (24)	0.97
			
Analgesia use			
Support person	65.3%	59.7%	0.18
Bath use	36.2%	32.3%	0.35
Epidural use	33.7%	32.8%	0.84
			
Birth weight (grams) (mean, SD)	3445 (451)	3512 (450)	0.11
			
Apgar scores > 7 at			
1 minute	221 (82)	149 (75)	0.12
5 minutes	351 (90)	167 (84)	0.68

Per cent may not add up to 100 due to missing values.

An a priori subgroup analysis was conducted excluding the group of the trial population who had lost their ability to choose their labour analgesia (after randomisation either due to a scheduled caesarean birth, an unplanned caesarean birth or an epidural due to medical reasons). There were no differences with regard to satisfaction with decision making once the women who had lost their options where excluded, (mean difference 0.063 (95%CI -0.99, 1.12), p = 0.90).

### Analgesia use, analgesia intentions

There were no significant differences between groups in regards to analgesia use. Non-pharmacological analgesics were used most frequently, and of these having a support person during labour was used by 63% of the total population. Nitrous oxide was the next most common analgesic (47%) and the most used pharmacological analgesic. Epidural analgesia was used by a third of the total group (33%).

There were no significant differences between analgesia intentions and use between the groups. There were however some significant differences in the total population between definite intentions to use an analgesic and actual use. The largest discrepancy between definite intentions to use an analgesic compared to actual use was for the three pharmacological analgesics. Amongst women who had no intentions to use nitrous oxide 40 per cent received it, and similarly just over twice the number of women who had definite intentions to use nitrous oxide(8.6%) used it 17.3% (χ^2 ^= 17.71, df = 5, p = 0.003). Only six per cent had definite plans to use epidural analgesia, however just over a third used it (37.8%), this includes 26.3% who were definitely not planning to use epidural analgesia (χ^2 ^= 23.1, df = 5 p = 0.003). Similarly for opioid analgesia only 1.5 per cent had definite plans to use it, however 22.2% per cent did use it (χ^2 ^= 26.3, df = 5, p < 0.0001). Overall women had used non-pharmacological analgesia more often than pharmacological analgesia and this was consistent with intentions. However definite intentions to use these methods also did not necessarily result in use. Only 75 per cent of women with definite intentions to have a support person actually did end up having one (χ^2 ^= 32.4, df = 5, p < 0.0001).

### Participation in decision making

There were significant differences between whether they had considered their care-providers opinion when making their labour analgesia decisions, the decision aid group were significantly more likely to report making the decision themselves after seriously considering their care-providers opinion compared to the pamphlet group who reported that they had made their labour analgesia decision by themselves (RR 1.28 95%CI (0.64, 0.95)).

## Discussion and conclusion

Pregnant women make labour analgesia decisions in a milieu of choice and anecdote that is on the whole devoid of evidence[19]. This is the first study to assist women with their labour analgesia decision making using an evidenced based decision aid. Other antenatal decision aids have been designed and implemented for specific pregnancy issues such as external cephalic version for breech presentation[11], and vaginal birth after caesarean section[10,13]. However, this is the first decision aid for a general pregnant population. Results from this trial demonstrated that a decision aid improves women's knowledge regarding labour analgesia without increasing their anxiety. Significantly, following intervention with the decision aid women were more informed of labour analgesia options, and were also more likely to feel that they had enough information to make their decisions and in labour considered the opinion of their care providers more often when making their labour analgesia decisions. Overall these results suggest that women who used the decision aid were more likely to make informed labour analgesia decisions.

Being knowledgeable and informed has been associated with being satisfied with the birth experience and with decision making [5], and previous research has shown that women want information that includes all the risks and benefits of analgesic options they are considering[5,25,26]. Results from this trial suggest almost all women want to be involved in their labour analgesia decisions, and antenatally most expected to make these decisions themselves. However, there were some postnatal differences between trial groups and who had participated in decision making that had occurred during labour. More women in the decision aid group considered their care-providers opinion during their decision making, and this was slightly more than had planned to antenatally. There were no differences in the high levels of satisfaction with decision making between groups which suggests that including care-providers in the decision making process even if unplanned does not reduce decision satisfaction. Conversely, not being involved with decision making has been shown to reduce satisfaction [5,27]. O'Connor suggests that general satisfaction with decision making could be more strongly affected by the practitioner relationship than the decision aid, and people find it psychologically comforting to say that they are satisfied rather than retrospectively doubt their own decisions[9]. Both of these factors could be partly responsible for the high levels of analgesia decision satisfaction in this trial. However regardless of the cause of the high satisfaction, results from this trial suggest that including care-providers in analgesia decision making, contrary to antenatal plans does not reduce satisfaction. These results will provide reassurance for care-providers who consider supporting women with decision making during labour an important aspect of their role[6].

The low decisional conflict scores in this population raise some interesting issues. Decision making literature suggests that those who will benefit from decision aids are in the active deliberation stage of decision making or in a stage where they are at least willing to consider or reconsider choices[9]. Prior to intervention approximately 80% of the total trial population were receptive to change, suggesting that they had not completely made their analgesic choices. However the decisional conflict scores were low, implying that women were experiencing only mild uncertainty regarding these decisions. Usually decisional conflict scores as low as 25 suggest follow-through of the decision. However, there was poor follow-through between definite analgesia intentions and use. Despite this, most women reported being satisfied with their decision making, our previous work in this area found that women were flexible with their choices and this flexibility may account for the discrepancy between analgesia plans and intentions.

Importantly, there are some practical and significant differences between the context of this decision aid and other contexts where decision aids have been used. Ideally decision aids are used in a setting where the users are at the point of decision making, this was not practical for this decision aid. Similar to the problems described by Barratt et.al [28], this decision aid involved a complex list of options which were not mutually exclusive, if one option was chosen it did not exclude other options, so each option needed to be considered, and these choices were required without full knowledge (e.g lack of knowledge of labour pain). Other research suggests that people do not reliably make decisions involving choice under uncertainty[29]. This is an important difference for the labour setting. As any decision made antenatally regarding labour analgesia includes a level of uncertainty. Importantly primiparous women do not have experience of labour pain and other factors such as labour staff preference and medical labour factors (for example length of labour) are known to influence labour analgesia use and these cannot be incorporated into an antenatal labour analgesia decision aid[30]. These important differences may preclude the appropriateness of decision follow-through as a measure of effectiveness in this context.

Women want labour analgesia information and our results show they utilised all available sources during their pregnancy. Women reported their social networks as the most important source of labour information, suggesting that labour analgesia decision making is largely informed by the experiences and knowledge of these networks, and not necessarily by risks and benefits. Antenatal classes were also rated as an important source of labour analgesia information, and these classes may be a useful method to use the decision aid, as the informal setting combined with the presentation by a childbirth educator may help with introducing evidence based information.

A strength of this study is the randomised controlled trial design using remote randomisation, high use of the interventions and high follow-up at primary outcome. The power of this trial to detect a difference in primary outcomes was further improved by the combination of the two decision aid arms. The low decisional conflict scores is of course an issue but does not affect the internal validity. It may be that we measured decisional conflict when women were not particularly conflicted regarding their labour analgesia, or that decisional conflict not be an appropriate measure for this decision aid in this context and population. Generalisability is the main limitation of this study. The population who participated in the trial were predominantly university educated, spoke English at home and (were limited to) primiparous. Women with self-assessed English insufficiency (to use the decision aid and or complete the questionnaires) were ineligible thereby limiting the generalisability. Other trials targeting low literacy populations have shown that decision aids designed for this group have positive results[31]. The effectiveness of the decision aid may also be greater amongst women who have higher levels of decisional conflict, and who are in the stages of decision making that are receptive to change. To get maximum benefit for users of this decision aid it may be beneficial to use it with women who are experiencing increased decisional conflict[9]. Of course a further limitation is that we have not done an economic evaluation of the decision aid and cost would be an important consideration for implementation, although the decision aid is now freely available on the internet.

Despite the limitations we believe that the decision aid is a beneficial tool for assisting women with their informed decision making and would also be useful for clinicians who can direct women to the website knowing that it is unbiased, evidence based information that does not increase anxiety. Finally, it is clear that women want to participate in their labour analgesia decision making. This decision aid improved women's labour analgesia knowledge, enabled them to involve their care-providers in analgesia decision making, did not increase anxiety, and maintained high satisfaction with analgesia decision making, overall providing women with the labour analgesia information they needed in an acceptable and well liked format.

## Competing interests

The authors declare that they have no competing interests.

## Authors' contributions

All authors contributed in the design and development of the project. CHRG was responsible for the analysis; NN was a contributor to the analysis. CHRG was primarily responsible for the manuscript with contributions from all authors. All authors have read and approved the final manuscript.

## Pre-publication history

The pre-publication history for this paper can be accessed here:

http://www.biomedcentral.com/1471-2393/10/15/prepub
